# Osteopathy—What Is It?

**Published:** 1897-08

**Authors:** 


### OSTEOPATHY—WHAT IS IT?

The latest aspirant for fame in the broad and well-trodden field of quackery is *Osteopathy*. Now that this noble science and art is attracting a good deal of unfavorable notoriety in the west, one naturally wonders what osteopathy is anyhow. Here we have all about it, in the last issue of the *Journal of Osteopathy*, defined etymologically, legally, historically and technically by the "discoverer" and great high priest of osteopathy, Dr. A. T. Still, of Kirksville, Mo.

"*Osteopathy*, [from] *osteon*, a bone, and *pathos*, suffering.

"*Legal:* A system, method, or science of healing.

"*Historical:* Osteopathy was discovered by Dr. A. T. Still, of Baldwin, Kan., 1874. Dr. Still reasoned that 'a natural flow of blood is health; and disease is the effect of local or general disturbance of blood—that to excite the nerves causes muscles to contract and compress venous flow of blood to the heart; and the bones could be used as levers to relieve pressure on nerves, veins and arteries.'

"*Technical:* Osteopathy is that science which consists of such exact, exhaustive, and verifiable knowledge of the structure and functions of the human mechanism, anatomical, physiological and psychological, including the chemistry and physics of its known elements, as has made discoverable certain organic laws and remedial resources, within the body itself, by which nature under the scientific treatment peculiar to osteopathic practice, apart from all ordinary methods of extraneous, artificial or medicinal stimulation, and in harmonious accord with its own mechanical principles, molecular activities, and metabolic processes, may recover from displacements, disorganizations, derangements and consequent disease, and regain its normal equilibrium of form and function in health and strength."

The *Journal of Osteopathy* before us is a unique publication and remarkable chiefly from containing a complete portrait gallery of the Still household, the various members of which, male and female, apparently represent at present the large and important American School of Osteopathy. The *Still-born* School of Osteopathy, of Kirksville, Missouri, confers a degree, Diplomate in Osteopathy (D.O.), upon those who master this intricate and remarkable science, and this, we are informed by the head of the house of Still, is the "technical and official designation of a graduate and practitioner in Osteopathy."

In short, osteopathy is the veriest nonsense and quackery that has disgraced an age already rich in many species of medical humbuggery. It is a fit associate for Christian science and orificial surgery. The so-called philosophy upon which these three miserable fakes base their existence is words, words, words, full of sound and fury, signifying nothing. One wonders where the boasted enlightenment of the Victorian era can be when osteopaths, Christian scientists, divine healers, faith curists and other voodoos can find willing victims.

We are pleased to see that the Governor of Illinois has wisely vetoed a late bill which passed the legislature of his State, which was designed to give a legal status to the "osteopathic school of practice"; and it is very much to be regretted that the Governor of Missouri did not have the good sense to do likewise. Any encouragement which these people receive from governors, legislatures, or the public, is a concession to ignorance and quackery.

